# Supplementary material for: Regulation of corneal stromal cell behavior by modulating curvature using a hydraulically-controlled organ chip array
Source: Nat Commun. 2025 Nov 12;16:9944. doi: 10.1038/s41467-025-64889-8 (PMC12612184; doi:10.1038/s41467-025-64889-8)
Supplement: Supplementary file 1 — Supplementary Information [file 41467_2025_64889_MOESM1_ESM.pdf]

Supplementary information

Regulation of Corneal Stromal Cell Behavior by Modulating Curvature Using a Hydraulically-Controlled Organ Chip Array

Minju Kim[1], Kanghoon Choi[1], David Krizaj[2], Jungkyu Kim[1,2]*

[1]Department of Mechanical Engineering, University of Utah, Salt Lake City, USA
[2]Department of Ophthalmology, University of Utah School of Medicine, Salt Lake City, USA

*Address correspondence to: Jungkyu (Jay) Kim
Department of Mechanical Engineering
The University of Utah
Salt Lake City, UT84112, USA
E-mail: jkim@mech.utah.edu

Supplementary information

Supplementary Note 1

The 3-, 6-, and 9-day time points shown in Supplementary Fig. 3 were strategically selected to monitor the temporal progression of stromal cell responses to curvature. Day 3 served as a pre-stimulation baseline, allowing sufficient time for cells to adhere, spread, and stabilize their initial phenotype on the substrate prior to curvature induction. Day 6 captured the early phase of the cellular response, during which mechanosensitive signaling pathways—such as α-SMA expression and focal adhesion assembly—begin to activate. Day 9 was designated to evaluate the later-stage responses, including sustained phenotypic transitions and extracellular matrix (ECM) remodeling processes. This timeline enabled comprehensive assessment of both immediate and progressive curvature-induced changes within a physiologically relevant 1-week period.

Supplementary Note 2

Strain and stress distributions were analyzed using COMSOL Multiphysics 5.5, as presented in Supplementary Figs. 5 and 6. Finite element analysis was performed using the Ogden hyperelastic material model[1], to simulate the curvature profile of the thin PDMS membrane. Supplementary Fig. 5 illustrates how net in-plane displacement varies across the three curvature conditions, providing insight into how geometric deformation imposes mechanical cues on adherent cells. As shown in Supplementary Fig. 5a, each curvature condition produced a characteristic displacement pattern composed of three distinct regions, demarcated by a shallow yellow transition zone. Based on these profiles, we defined three spatial regions—center, slope, and edge—corresponding to areas of different displacement magnitudes

(Supplementary Fig. 5b). These regions were identified by thresholding the yellow boundary displacement at 0.03 mm for low curvature, 0.045 mm for medium curvature, and 0.10 mm for high curvature. Accordingly, the center region was defined as the inner 1 mm diameter area, the slope as the annular zone between 1 mm and 3.5 mm, and the edge as the outermost area beyond 3.5 mm in diameter.

Supplementary Note 3

To culture cells on PDMS surfaces, various extracellular matrix (ECM) proteins, including fibronectin, gelatin, and collagen, have been employed[2]. However, when these ECMs are adhered to PDMS via physisorption, the resulting weak interactions often lead to unstable cell layers under mechanically stimulating conditions[3]. In preliminary tests, cells seeded on uncoated PDMS substrates exhibited poor attachment and frequent detachment, particularly under curvature-induced deformation, likely due to weak physisorption of ECM proteins and hydrophobicity of the PDMS surface. To address this, we evaluated polydopamine (PDA) treatment to enhance ECM adhesion. Supplementary Fig. 7 shows the viability of three stromal cell types cultured on collagen-coated PDMS surfaces following polydopamine (PDA) treatment. Cell viability was assessed using the Cell Counting Kit-8 (CCK-8, 96992, Sigma-Aldrich, USA) across five dopamine concentrations (0.005, 0.01, 0.05, 0.25, and 0.5% w/v). A 10:1 ratio of CCK-8 solution (20 μL) was added to each well of the array chips containing cultured cells in 200 μL of medium, followed by a 4-hour incubation at 37 °C. After incubation, 100 μL of the supernatant was transferred to a 96-well plate, and absorbance was measured at 450 nm using an ELISA reader (BioTek Epoch2, BioTek Instruments Inc., Winooski, VT, USA). Cell viability remained high at 0.005% and 0.01% dopamine concentrations for all cell types, whereas

concentrations ≥0.05% significantly reduced viability. Supplementary Fig. 7d shows CellTracker-stained images of viable cells, where background particle aggregation became prominent at 0.25% and 0.5% w/v. Such aggregation is a known outcome of excessive dopamine polymerization[6]. To minimize these effects while ensuring stable cell adhesion, 0.01% w/v dopamine was selected as the optimal concentration for PDA coating following oxygen plasma treatment.

Supplementary Note 4

Corneal stromal cells were seeded onto a PDA-assisted collagen-coated curvature array chip by applying a concentrated droplet at the center of each well to assess curvature-driven cell growth dynamics from apex to periphery. A bioprinter was used to dispense approximately 3 μL of cell suspension per well, yielding an average initial seeding area of 1.93 ± 0.11 mm² ($n = 5$), which corresponded to approximately 2–3% of the total curved surface area (Supplementary Fig. 8). By accounting for the differential proliferation rates of keratocytes, fibroblasts, and myofibroblasts, we adjusted the initial seeding densities to ensure comparable final cell coverage across all three cell types by day 9 (D9). As shown in Supplementary Fig. 8c, corneal keratocytes initially exhibited a larger seeding spread compared to fibroblasts and myofibroblasts on day 0. However, by employing two tailored seeding concentrations based on cell-specific morphology and proliferation potential, complete confluence over the entire 8-mm diameter circular region was achieved by D9 (Fig. 2a). This result validated both the consistency of the seeding method and its utility in quantifying curvature-mediated differences in cell proliferation.

Supplementary Note 5

Nuclear staining with Hoechst enabled visualization of cell localization within the central region of the 8-mm circular area, as shown in Supplementary Fig. 8a. As quantified in Supplementary Fig. 8b, a seeding concentration of 1×10^6 cells/mL resulted in a cell coverage density of 42,129 ± 3,005 cells/cm², whereas a lower concentration of 2.5×10^5 cells/mL yielded a density of 9,010 ± 2,398 cells/cm². Given that quiescent corneal keratocytes exhibit lower proliferation rates compared to their activated counterparts (corneal fibroblasts and myofibroblasts)[7], keratocytes were seeded at the higher concentration (1×10^6 cells/mL), while activated stromal cells were seeded at 2.5×10^5 cells/mL to ensure comparable final cell densities by day 9.

Supplementary Note 6

Quiescent corneal keratocytes exhibited a characteristic dendritic morphology[4], forming long and shallow bundle of branches (Supplementary Fig. 10a). Upon activation, keratocytes transitioned into fibroblasts and myofibroblasts. Fibroblasts displayed an elongated morphology [5] , lacking dendritic extensions and showing close cell–cell adhesion (Supplementary Fig. 10a). Myofibroblasts adopted a stellate shape with thicker branches than quiescent keratocytes[6]. Immunofluorescence staining for aldehyde dehydrogenase (ALDH3A1) and α-smooth muscle actin (α-SMA) confirmed the phenotypic identity of each cell type. As shown in Supplementary Fig. 10a and c, keratocytes expressed high levels of ALDH3A1, whereas fibroblasts and myofibroblasts exhibited little to no ALDH3A1 signal. Conversely, α-SMA expression was nearly undetectable in keratocytes but markedly increased in fibroblasts and myofibroblasts (Supplementary Fig. 10a, d). Notably, myofibroblasts formed prominent stress fibers, a hallmark

of their highly contractile phenotype[7]. Focal adhesion formation is closely linked to cellular contractility and α-SMA expression in fibroblasts and myofibroblasts[8], as well as to stiffness-mediated differentiation in keratocytes[9]. Supplementary Fig. 10b shows F-actin and vinculin staining across the three cell types. Activated fibroblasts and myofibroblasts exhibited thicker F-actin bundles compared to keratocytes. In addition, keratocytes showed low vinculin expression, while fibroblasts and myofibroblasts displayed significantly higher levels of vinculin (Supplementary Fig. 10b, e), further supporting their activated, adhesion-dependent phenotypes.

Supplementary Note 7

Deionized water (DIW) used for hydraulic actuation was pre-warmed to 37 °C prior to injection, and all handling was performed under sterile conditions using biosafety cabinet protocols. During DIW injection, DIW was introduced through the inlet port while air was expelled via the outlet port of each hydraulic chamber using a 30G needle connected to a 1 mL syringe (Fig. 1b). Inserting syringes into both inlet and outlet ports enabled effective air removal and liquid filling, thereby achieving the desired curvature. Due to the self-recovery properties of PDMS, needle punctures at the ports were sealed without leakage, allowing the curvature to be stably maintained over time. The thick PDMS reservoir layer provided structural support against delamination under hydraulic pressure, ensuring curvature stability. The relationship between injection volume and curvature was determined by capturing angle profiles during DIW injection using a stereomicroscope coupled with a Ximea CCD camera (model MQ042RG-CM, Ximea), as shown in Supplementary Fig. 2. The chamber was pre-filled with 45 μL of DIW, followed by continuous injection at a rate of 60 μL/min. The resulting images were analyzed using ImageJ to quantify curvature angles.

Supplementary Note 8

The human cornea has an average radius of curvature of approximately 7.80 mm, corresponding to ~43 diopters (D) based on the formula K (diopters)=337.5/radius, where K is the keratometric diopter. Clinically, corneal curvature less than 36 D is indicative of cornea plana, while curvatures exceeding 48 D are associated with keratoconus or keratoglobus disorders[1, 2, 3]. To emulate the anatomical variation in corneal curvature, we designed three curvature profiles with radii of 6 mm (high), 8 mm (medium), and 10 mm (low), approximating pathological and physiological conditions relative to the average radius of 7.8 mm. Each curvature was defined using a fixed chord length (c) of 8 mm, and the corresponding arc height (h) and segment angle (α) were derived using standard circular segment equations: $R = (h/2) + (c^2/8h)$, $\alpha = 2\ \sin^{-1}(c/2R)$, and $c = 2R\ \sin(\alpha/2)$, $s = 2\Pi r\ (\alpha/360°)$, where R is the radius, α\alphaα is the angle, h is the segment height, and c is the chord length (Supplementary Fig. 19, Supplementary Table 1). Based on these calculations, the high curvature condition (6 mm radius) represented keratoconus/keratoglobus, with a curvature of 56.25 D and an angle of 20.91°. The medium curvature (8 mm radius) corresponded to a physiologically normal cornea at 42.18 D and 15°, while the low curvature (10 mm radius) modeled cornea plana, with a curvature of 33.75 D and an angle of 11.79°.

Supplementary Note 9

The samples were imaged using a tiled imaging application to capture a comprehensive view of the entire area. After fixation, all images were captured in the planar state, with both brightfield and fluorescent images taken from a quadrant of the 8 mm diameter circle. The cells that migrated farthest from the center or closest to the edge of the 8 mm circle—where at least

three cells were present—were selected to define the edge line of the cell contour. The images were pre-processed using thresholding and binary options in ImageJ prior to outlining. The pre-processed images of the curvature array chip were analyzed using the outline function in ImageJ. The outlines of the cells were manually adjusted using shape tools with a size of 200 pixels to eliminate any unsuitable areas. The modified outlines were saved along with their respective coordinates. After obtaining the saved outlines, the coordinates of the cell contours were plotted based on the center of each circle.

The cell coverage radius was evaluated based on the measured cell outlines along the 45° direction. The changes in the outline observed along this direction on day 3, day 6, and day 9 after droplet seeding were converted into cell coverage area. This coverage rate analysis was performed using the curvature surface area formula, under the assumption that the entire cell-covered area maintained a uniform circular shape. The calculated cell coverage areas between day 3 and day 6 were then converted into cell coverage rates. We hypothesized that the cells proliferated in a perfect circle (πr^2), and calculated the proliferated area using the length (L) from the center to the outermost cells in the 45° direction. The radius of the edge line was measured using ImageJ, and the resulting circular area was adjusted to reflect the curvature surface area. The estimated radius (L) on the flat surface was converted to curvature surface area to accurately determine the proliferation rates on different curvatures (low, medium, and high). This conversion was necessary due to the varying surface areas associated with each curvature, whereas the flat condition represented a uniform 8 mm diameter circle.

The curvature surface area was calculated using the formula: surface area = $2\pi rh$, where $h = L\cdot\tan\theta$. Each curvature had a different tangent value and radius. For low curvature, $\tan\theta$ = 0.2075 with a radius of 10 mm; for medium curvature, $\tan\theta$ = 0.268 with a radius of 8 mm; and

for high curvature, $\tan\theta = 0.383$ with a radius of 6 mm. Specific values for each curvature condition are provided in Supplementary Table 1.

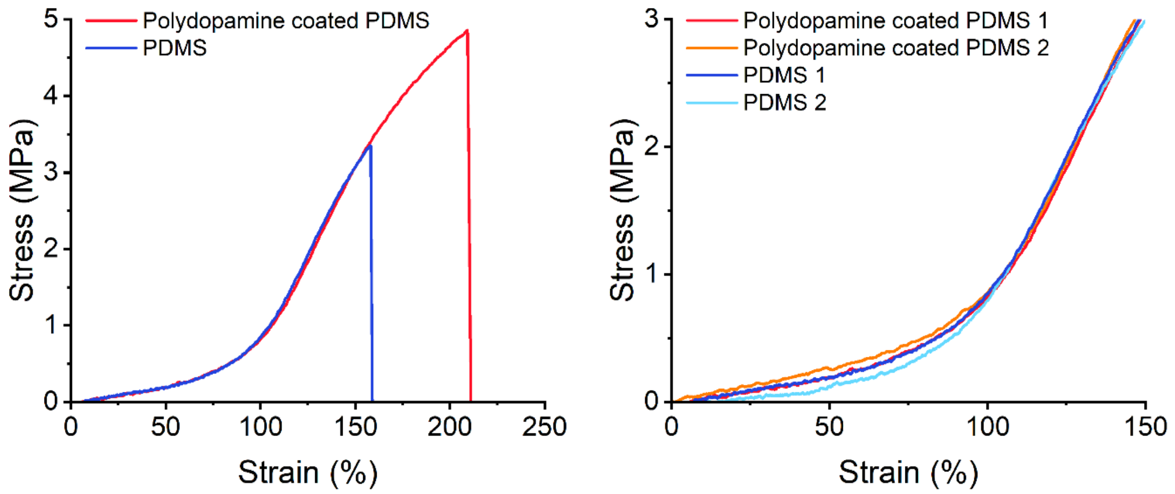

Supplementary Figure 1. Elastic modulus of polydopamine coated PDMS compared to non-coated PDMS. The PDMS film with 10:1 component was coated with 0.01% w/v dopamine concentration for overnight. The elastic modulus of polydopamine coated PDMS and PDMS was evaluated using tensile strength tester (5969 Dual column testing system, Instron). Polydopamine coated PDMS and non-coated PDMS showed similar elastic modulus values.

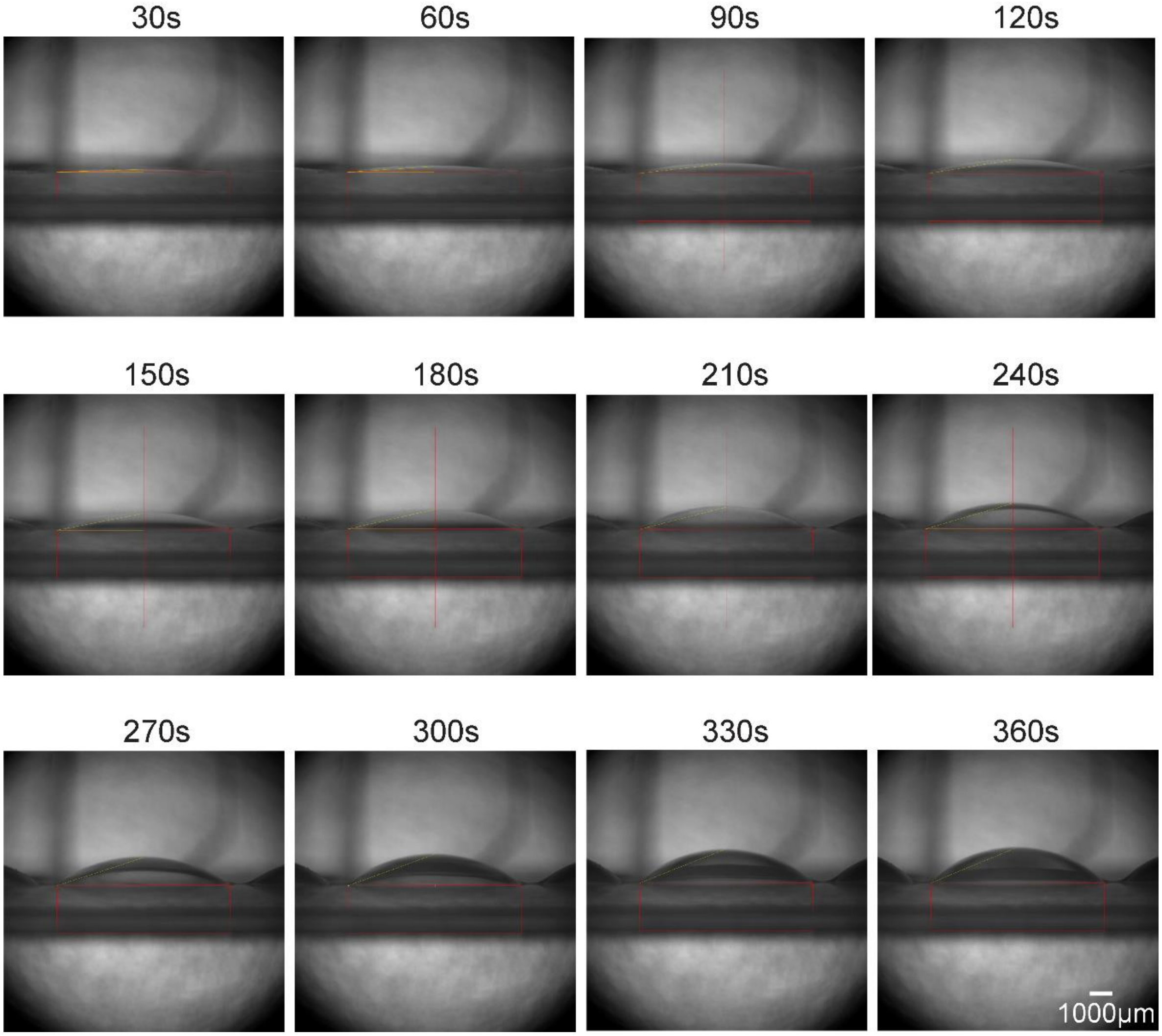

Supplementary Figure 2. Time-lapse images of the angle profiles during fluid injection in the hydraulic channel by volume dependent. The side view images were captured by stereo microscopy with Ximea CCD camera (Ximea, MQ042RG-CM). The angle of the curvature was analyzed depending on volume per second using imageJ software. The initial point for the experiment was set at 45 μL at 0 seconds, considering the volume of the hydraulic channel. This volume was chosen as the starting point to ensure proper functioning and calibration of the experimental setup.

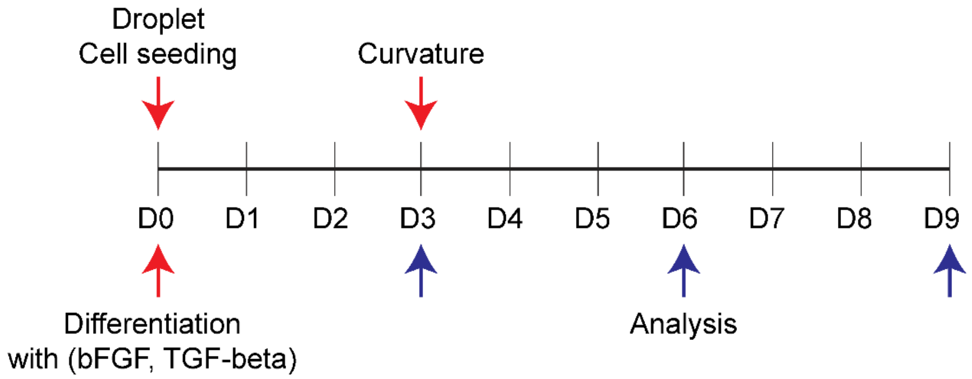

Supplementary Figure 3. Schematical illustration of the experimental timeline. The initial point where the cornea keratocytes are seeded onto the center of the substrate on D0. The cells are seeded and allowed to adhere and stabilize. The cornea keratocytes are treated with bFGF and TGF-beta to induce activation and differentiation on day 0- day 9 (D0-D9). The developed cornea keratocytes are analyzed to assess the effects of the bFGF and TGF-beta treatment on day 3 (D3). The developed cornea keratocytes are subjected to curvature treatment on day 3- day 9 (D3-D9). The three types of cells are analyzed to evaluate the impact of the curvature on their behavior and phenotype on D6 and D9.

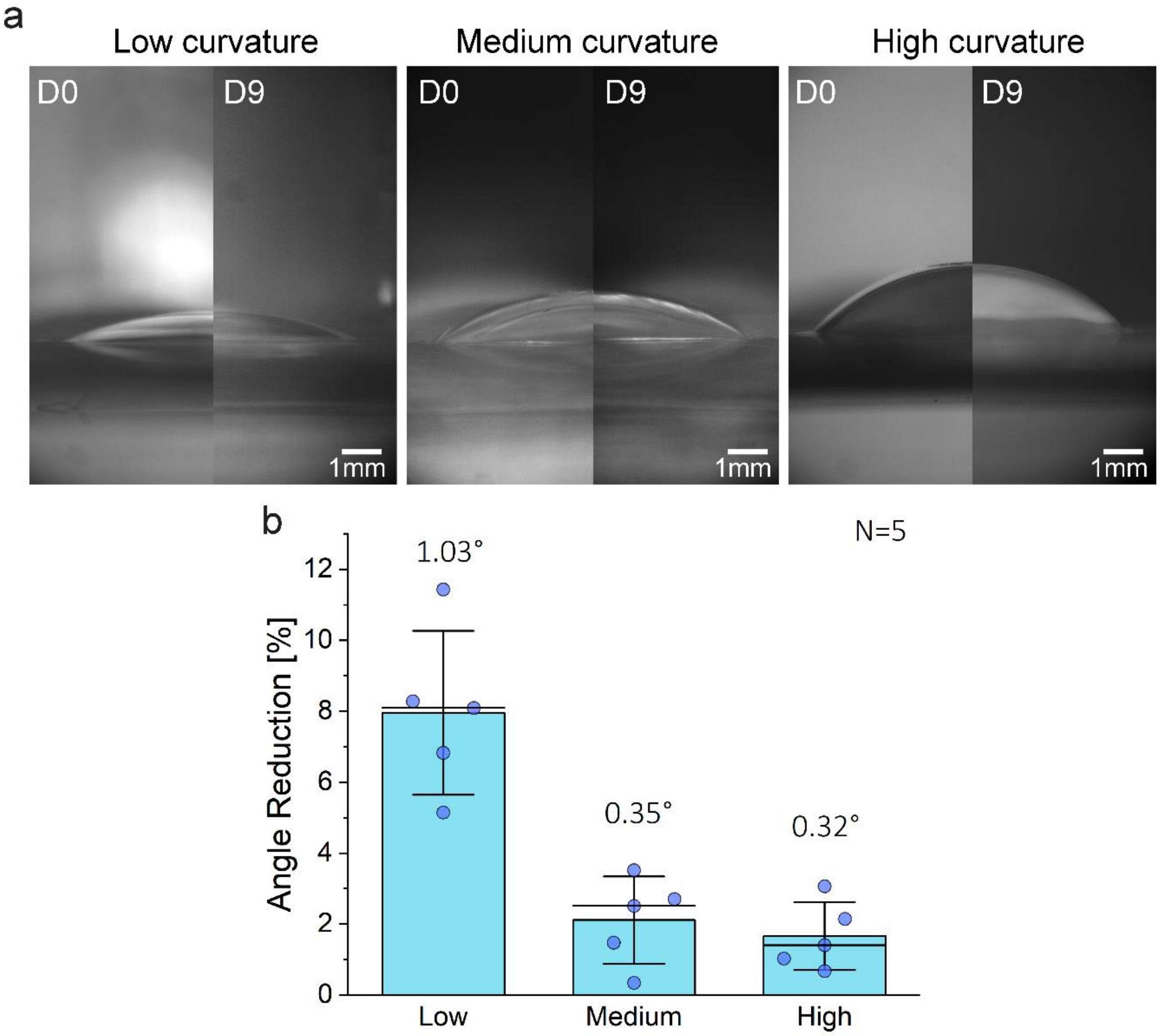

Supplementary Figure 4. Stability of curvature profiles over 9 days. a) Side-view images of the chip showing maintained curvature on day 0 (D0) and day 9 (D9) across all curvature levels. b) Quantification of curvature angle reduction (%) between D0 and D9. Bar plots indicate mean ± standard deviation (s.d.), with ● representing individual data points (N = number of independent replicates, unit = chip).

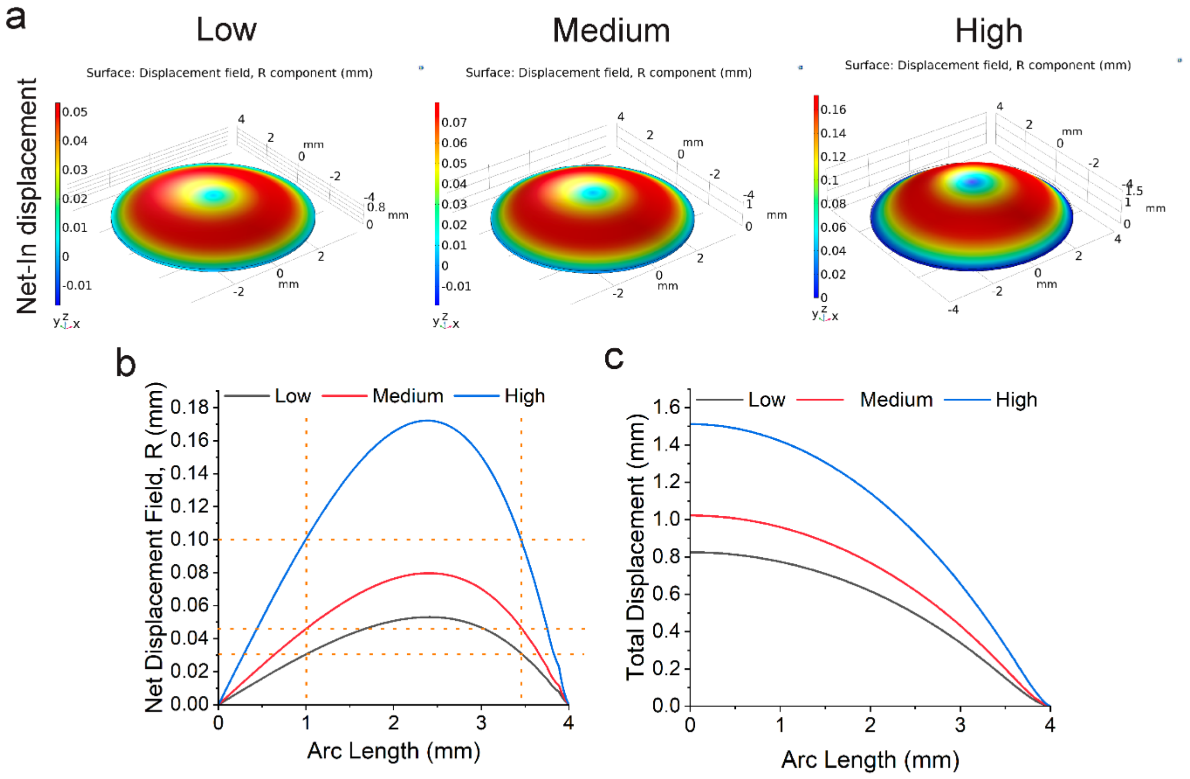

Supplementary Figure 5. COMSOL Multiphysics 5.5 deformed curvature surface simulation. a) The net-in plan displacement profile was analyzed by COMSOL Multiphysics 5.5 when hydraulic pressure was injected into the hydraulic chamber. The net-in plan displacement profiles were evaluated by three different curvatures: low, medium, and high. **b)** Net-in plan displacement determines the localized areas: center, slope, and edge. **c)** The total height displacement was quantified on the plot.

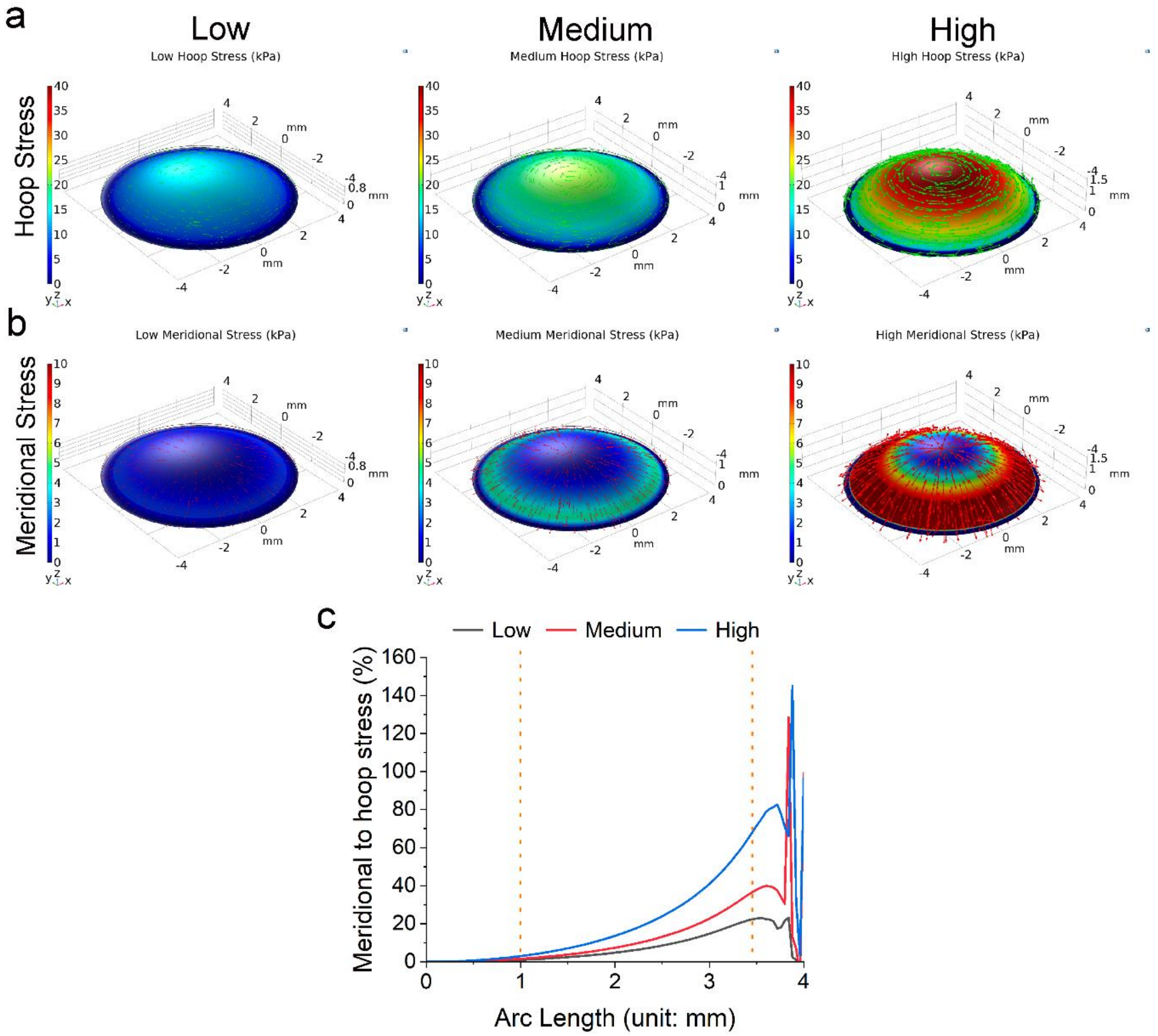

Supplementary Figure 6. Deformed curvature surface stress profiles with meridional and hoop stress. a) COMSOL Multiphysics 5.5 stress profiles by injecting hydraulic pressure into a hydraulic chamber. Hoop stress profiles include the vector field of stress depending on three different curvatures: Low, Medium, and High. **b)** Meridional stress profiles include the vector field of stress. **c)** The percentage of the hoop stress over the meridional stress depending on three different curvatures.

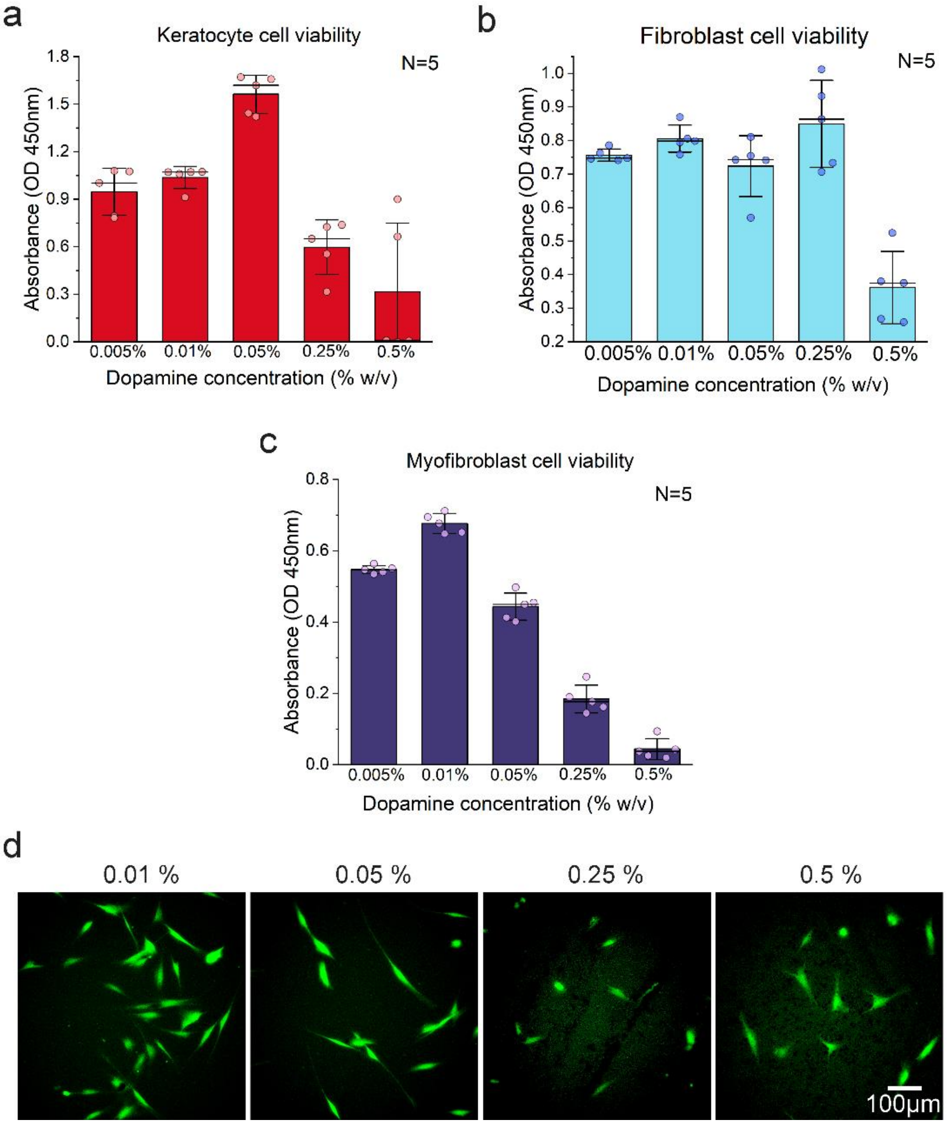

Supplementary Figure 7. Cell viability on collagen-coated PDMS treated with polydopamine. To evaluate the cell viability on dopamine and collagen-coated PDMS substrates, different concentrations of dopamine (0.005%, 0.01%, 0.05%, 0.25%, and 0.5% w/v) were used. The cell viability was assessed with Cell Counting Kit-8 by measuring the absorbance after 4 hours at 450nm (OD 450nm). **a-c)** The cell viability of quiescent form cornea keratocyte, activated formed cornea fibroblast, activated formed cornea myofibroblast were evaluated for different dopamine concentrations. Bar plots indicate mean ± standard deviation (s.d.), with ● representing individual data points (N= independent biological replicates). **d)** The 0.01% w/v dopamine concentration was determined with 50ug/ml collagen concentration due to the observed cell toxicity associated with higher concentrations of polydopamine.

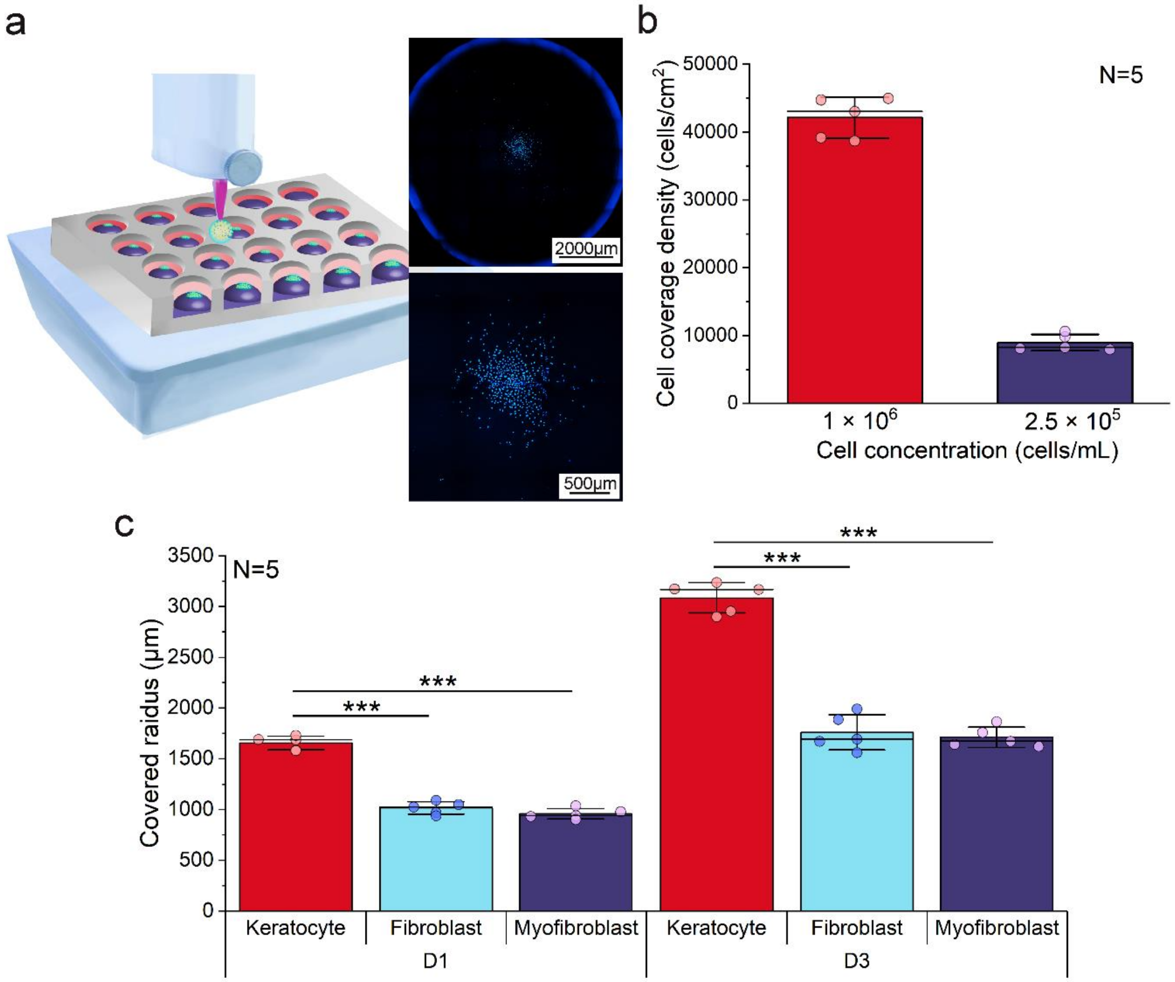

Supplementary Figure 8. Droplet cell seeding and coverage. a) Illustration of the bioprinter to demonstrate the droplet cell seeding on multi-well curvature chip. The Hoechst-stained image was used to analyze the cell coverage density and the difference between the two densities. **b)** Approximately four times larger cell coverage of $1×10^6$ cell concentration than 2.5×105 cell concentration. Bar plots indicate mean ± standard deviation (s.d.), with ● representing individual data points (N = independent biological replicates). **c)** The analysis of cell coverage area under different induction media on Day 1 and Day 3: the proliferation patterns of the three distinct cell types continued up to Day 3 with a similar pattern before the curvature application. Bar plots indicate mean ± standard deviation (s.d.), with ● representing individual data points (N = independent biological replicates). Statistical significance was assessed by two-sided one-way analysis of variance (ANOVA) followed by Scheffé's post hoc test (*P < 0.05, **P < 0.01, ***P < 0.001)

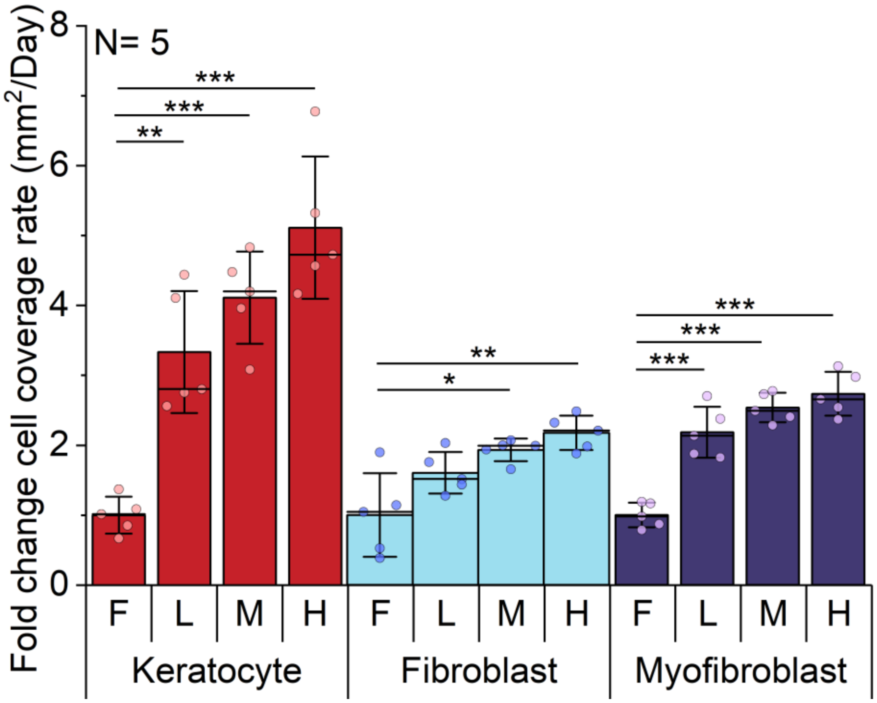

Supplementary Figure 9. Effect of curvature on fold change in cell coverage rate of corneal stromal cells. Fold change in cell coverage rate between flat and curved conditions (low, medium, and high curvature) was calculated for keratocytes, fibroblasts, and myofibroblasts between day 3(D3) and day 6 (D6). Bar plots indicate mean ± standard deviation (s.d.), with ● representing individual data points (N = independent biological replicates). Statistical significance was assessed by two-sided one-way analysis of variance (ANOVA) followed by Scheffé's post hoc test (*P < 0.05, **P < 0.01, ***P < 0.001)

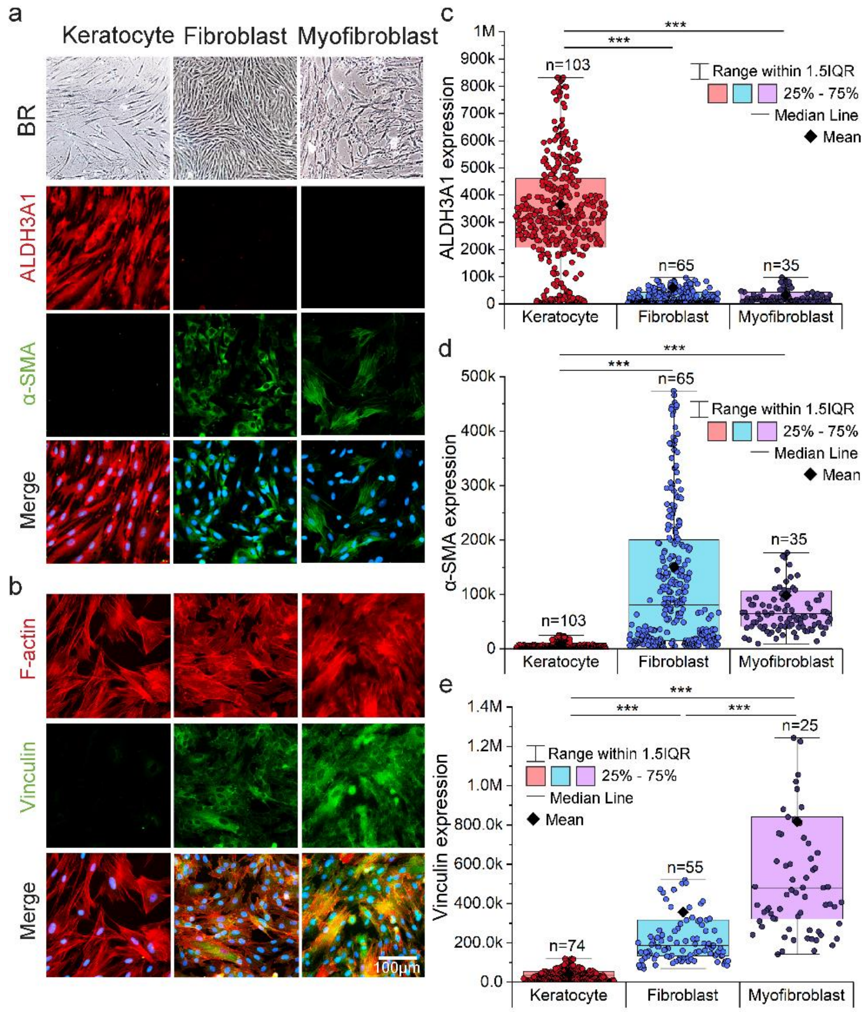

Supplementary Figure 10. Morphological and phenotypic characteristics of cornea stromal cells (keratocytes, fibroblasts, and myofibroblasts). a) Bright-field images highlighting the respective cell morphologies: dendritic-shaped keratocytes, spindle-shaped fibroblasts, and stellar-shaped myofibroblasts, along with immunostaining images for ALDH3A1 (red) and α-SMA (green), with nuclei counterstained by Hoechst (blue). **b)** Immunostaining images of f-actin and focal adhesion for all cornea stromal cells. **c-d)** A comparative analysis of ALDH3A1 and α-SMA for each cell type. **e)** Relative vinculin intensity: higher vinculin expression in myofibroblasts and fibroblasts than keratocytes. ALDH3A1, aldehyde dehydrogenase; SMA, smooth muscle actin. Box plots indicate the 25th–75th percentiles (box), with whiskers extending to the minimum and maximum values within 1.5× the interquartile range (IQR). The center line denotes the median, ◆ indicates the mean, and ● represent individual data points (n = number of analyzed images). Statistical significance was assessed by two-sided one-way analysis of variance (ANOVA) followed by Scheffé's post hoc test (*$P < 0.05$, **$P < 0.01$, ***$P < 0.001$)

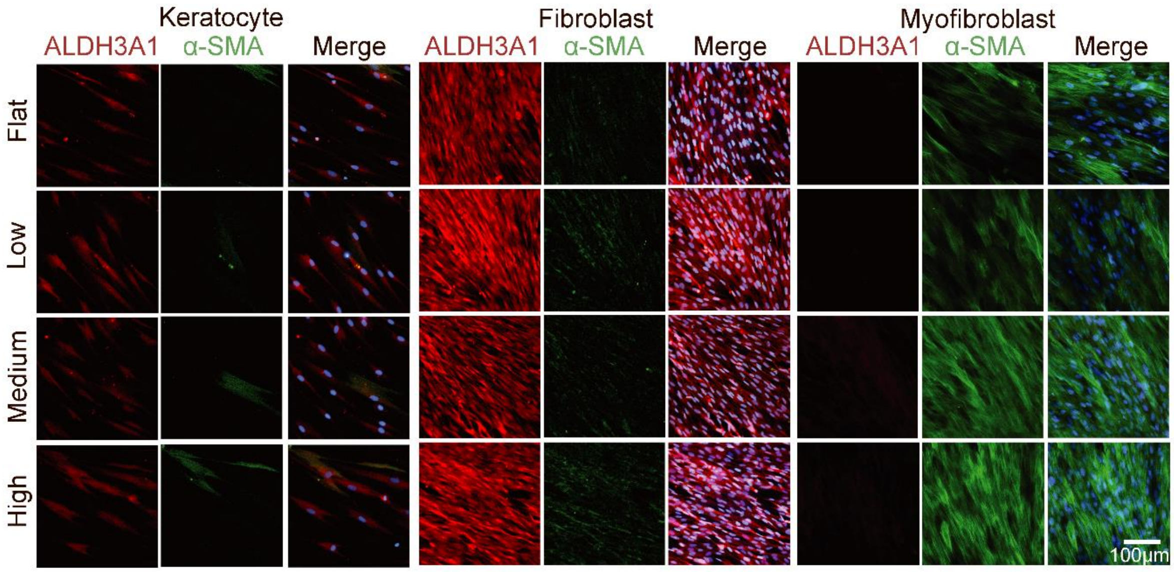

Supplementary Figure 11. ALDH3A1 and α-SMA expressions of all stromal cells were analyzed on Day 9 (6 days under curvature). Curvature was first introduced via immunofluorescence staining with cell phenotypes related markers. 40 × magnification images of all cell types showed modifying cell phenotypes compared to flat by expressing the high level of α-SMA expression on high curvature even in quiescent formed corneal keratocytes.

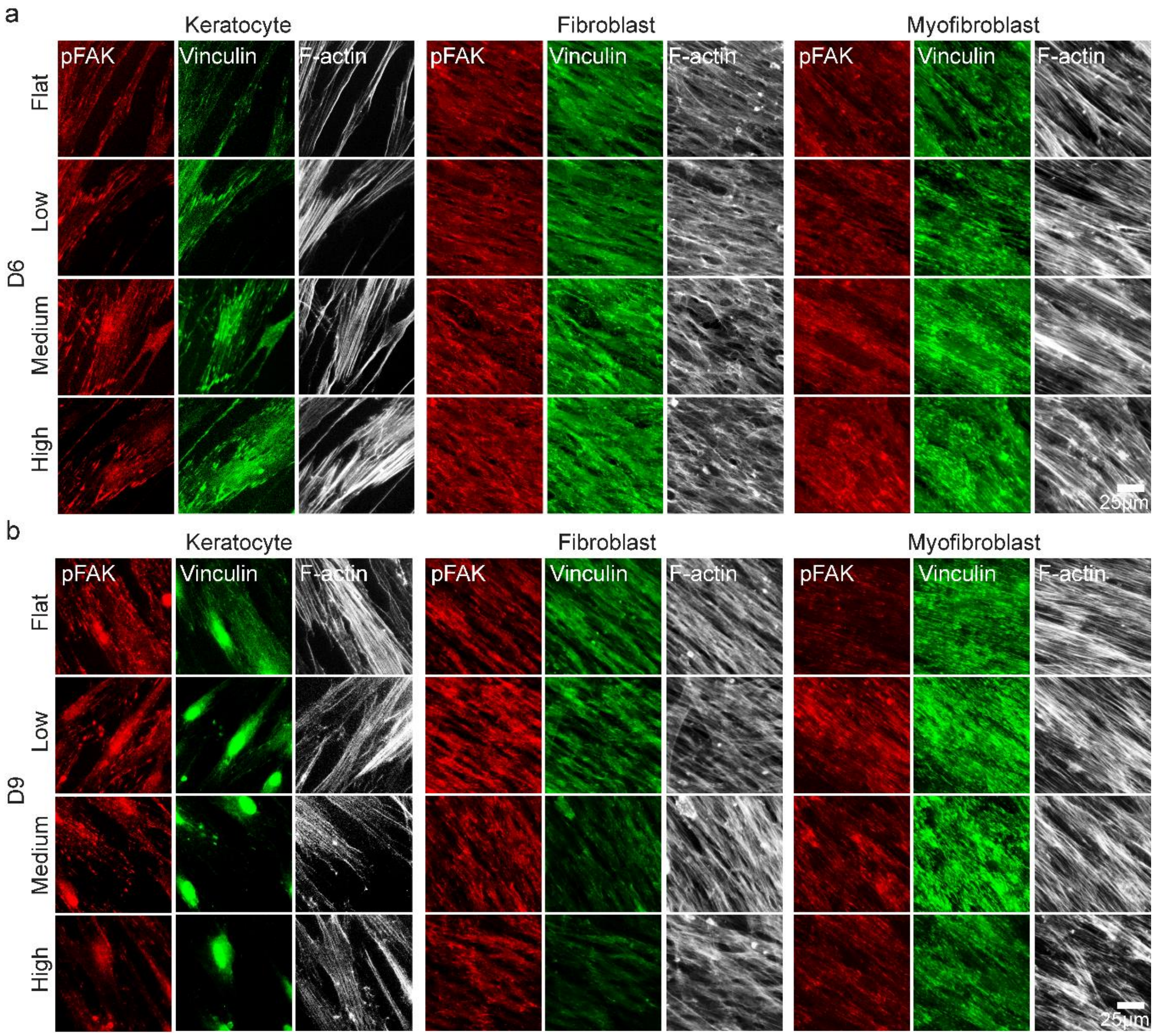

Supplementary Figure 12. Immunostaining image of focal adhesion markers pFAK and Vinculin in corneal stromal cells under curvature. a) Representative fluorescence images (×120) show pFAK (red) and Vinculin (green) expression at day 6 (D6) for keratocytes, fibroblasts, and myofibroblasts cultured on flat, low, medium, and high curvature substrates. At D6, focal adhesion formation increased with curvature across all cell types. **b)** By day 9 (D9), however, only myofibroblasts retained elevated pFAK and Vinculin expression, while keratocytes and fibroblasts exhibited reduced focal adhesion.

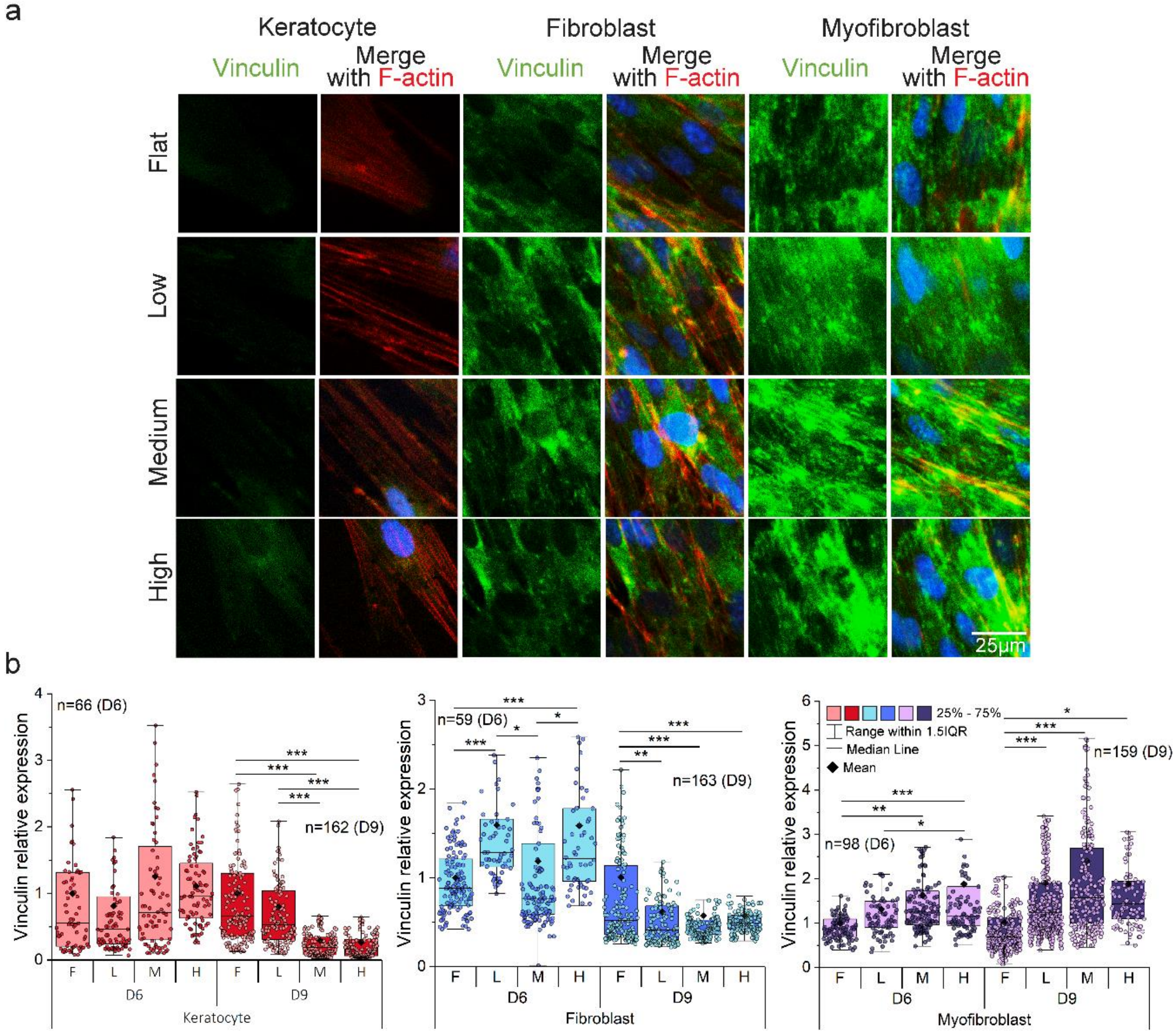

Supplementary Figure 13. Curvature-induced focal adhesion expression of cornea stromal cells. a) Vinculin expression on day 6 (D6) of all cell types on various curvatures (Green: Vinculin, Red: F-actin, Blue: Nuclei). **b)** Quantitative analysis of the vinculin mean intensity on various curvatures on D6 and day 9 (D9). Box plots indicate the 25th–75th percentiles (box), with whiskers extending to the minimum and maximum values within 1.5× the interquartile range (IQR). The center line denotes the median, ◆ indicates the mean, and ● represent individual data points, n = number of analyzed images. Statistical significance was assessed by two-sided one-way ANOVA followed by Scheffé's post hoc test (*P < 0.05, **P < 0.01, ***P < 0.001)

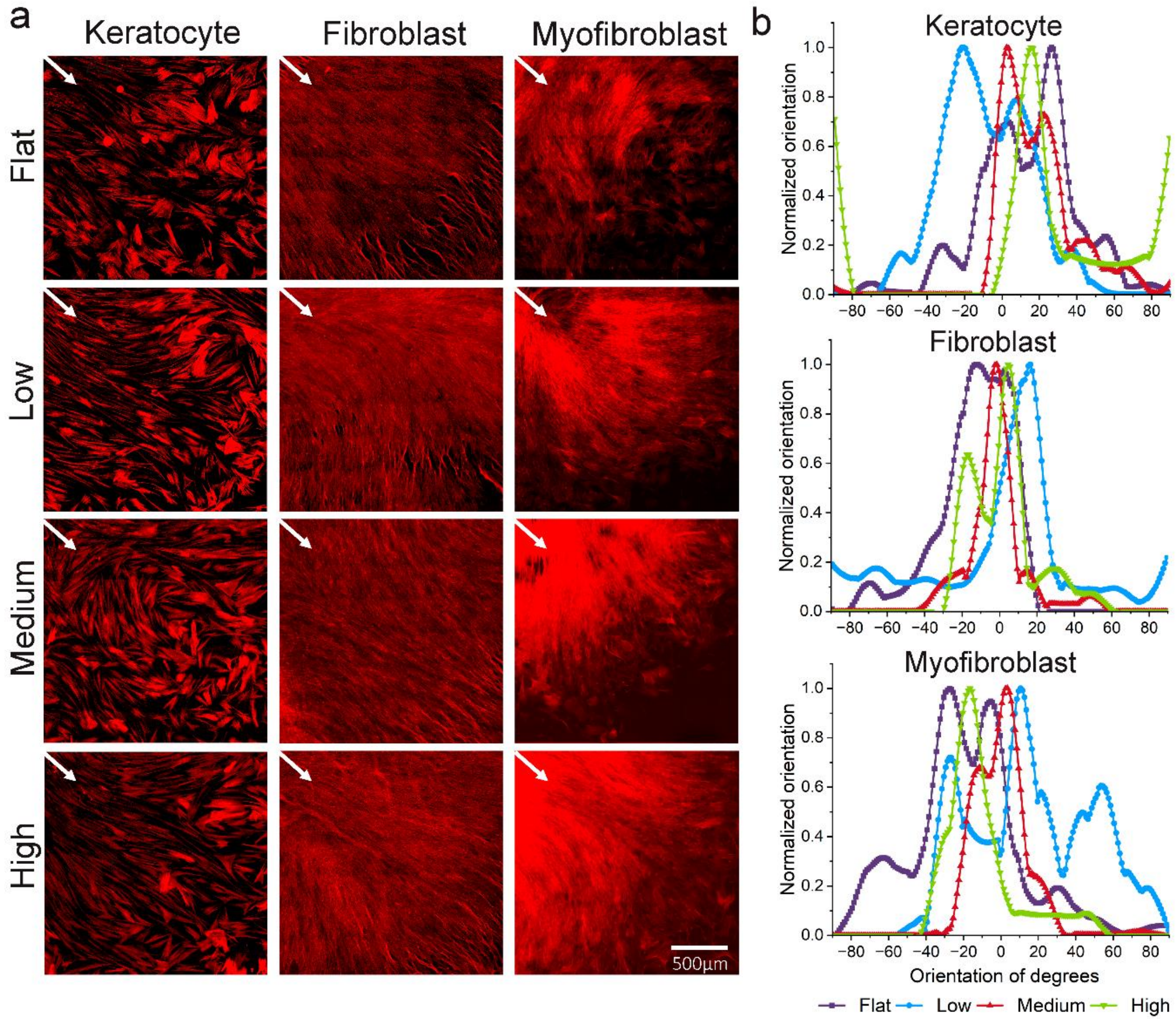

Supplementary Figure 14. The curvature-influenced cell orientations of cornea stromal cells. F-actin images for the analysis of cell orientation and normalized distribution. **a)** The part of tile f-actin images that were selected on the same location (1982μm×1982μm) in 45 directions showed a significant difference concerning the curvature. Cornea keratocyte showed random cell orientation compared to fibroblast and myofibroblast, but high curvature showed a relatively aligned cell orientation for all cell types. Especially, fibroblast and myofibroblast exhibited aligned cell organization on medium and high curvature. **b)** Normalized distribution of cell orientation showed the medium and high curvature influence the cell orientation by expressing the shallow and significant peak compared to low curvature and flat.

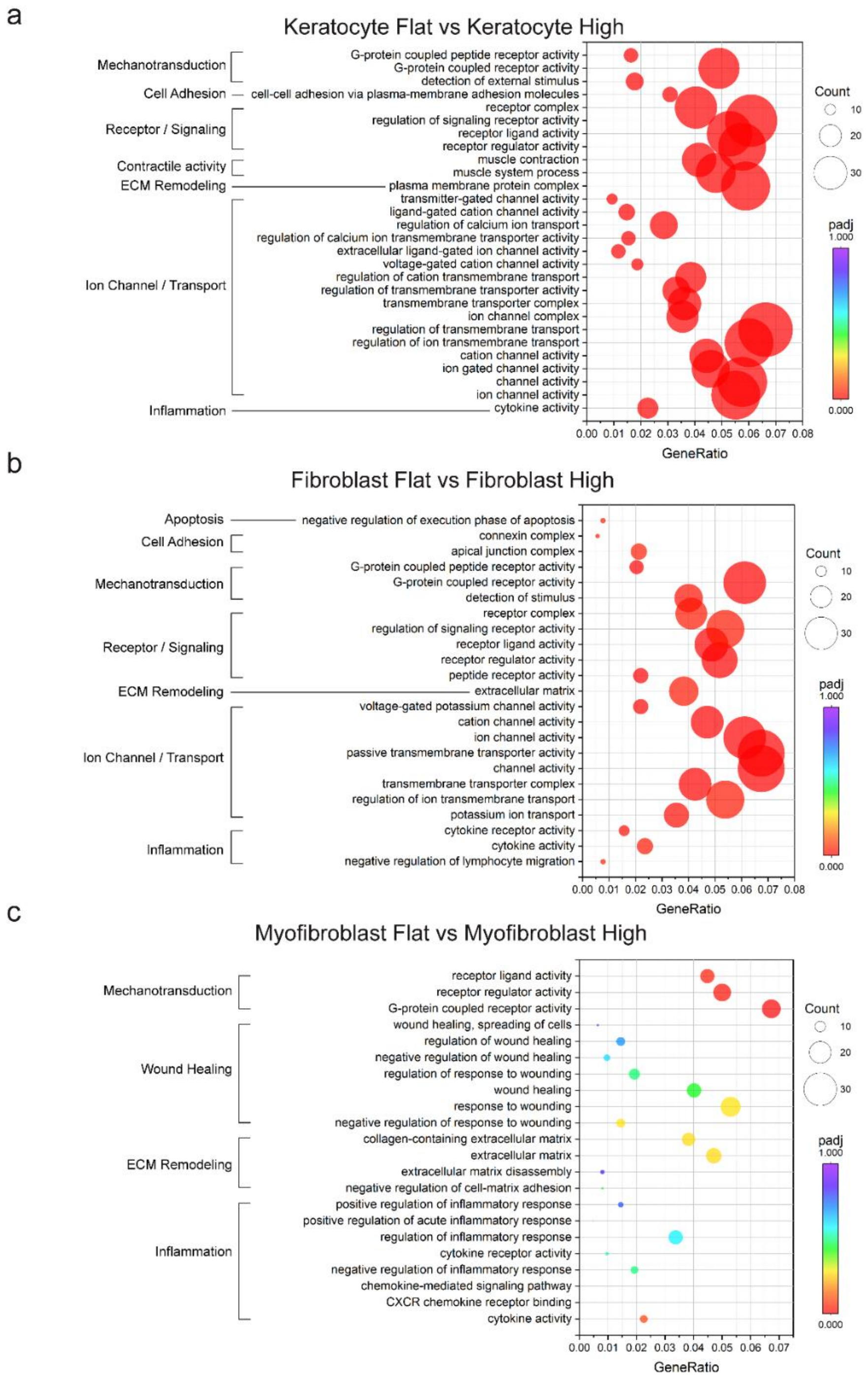

Supplementary Figure 15. Gene ontology (GO) enrichment analysis of curvature-responsive genes in corneal stromal cells. a) GO terms significantly enriched in keratocytes under high curvature compared to flat conditions. **b)** GO terms enriched in fibroblasts cultured under the same conditions. **c)** Enriched GO terms in myofibroblasts reveal distinct curvature-responsive biological processes and signaling pathways.

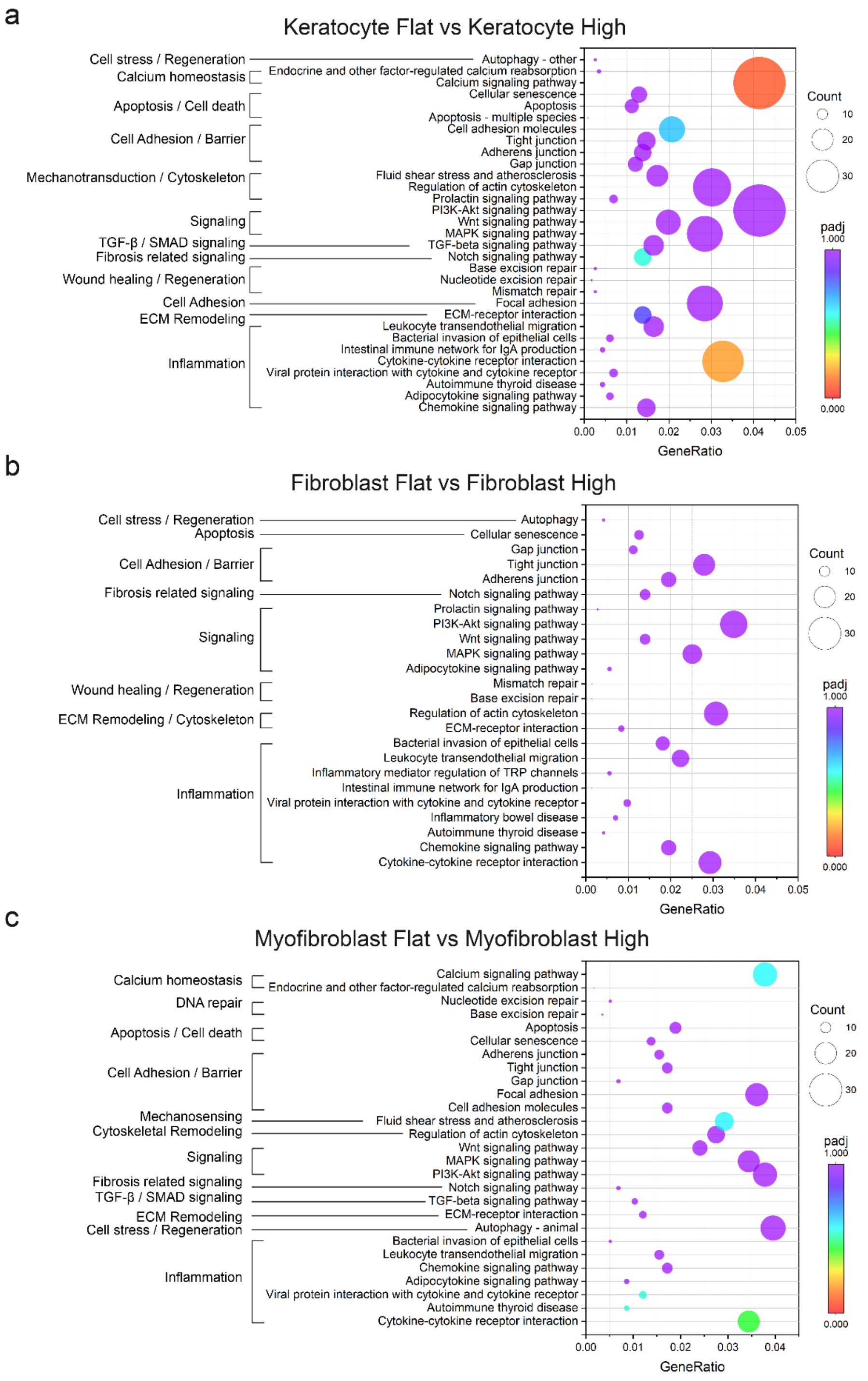

Supplementary Figure 16. KEGG pathway enrichment analysis of curvature-responsive genes in corneal stromal cells. a) KEGG pathways significantly enriched in keratocytes under high curvature compared to flat conditions. **b)** Enriched KEGG pathways in fibroblasts exposed to high curvature. **c)** KEGG pathway enrichment in myofibroblasts highlights distinct curvature-responsive biological processes and signaling mechanisms.

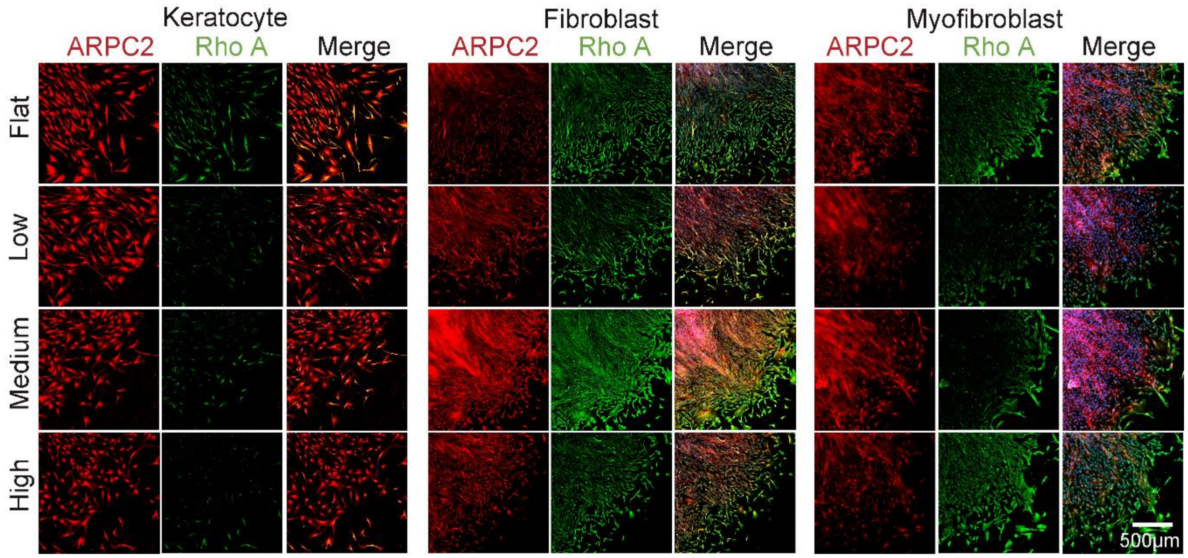

Supplementary Figure 17. The curvature-influenced Rho and Rac activity. ARPC2 and Rho A markers related to the Rho and Rac activity pathway were stained for all cell types on day 6(D6) to evaluate the curvature effect from collective migration. The expression on Rho A for cornea keratocytes from curvature showed a distinct decrease trend compared to flat.

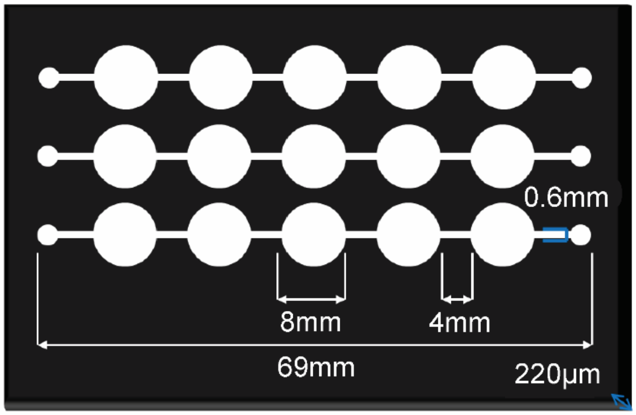

Supplementary Figure 18. The design of hydraulic chamber with microfluidic channels. The dimensions of the hydraulic chamber for 4×5 curvature array chip; 0.6 mm width, 220μm height, and 69 mm length containing 8 mm diameter wells with 4 mm intervals.

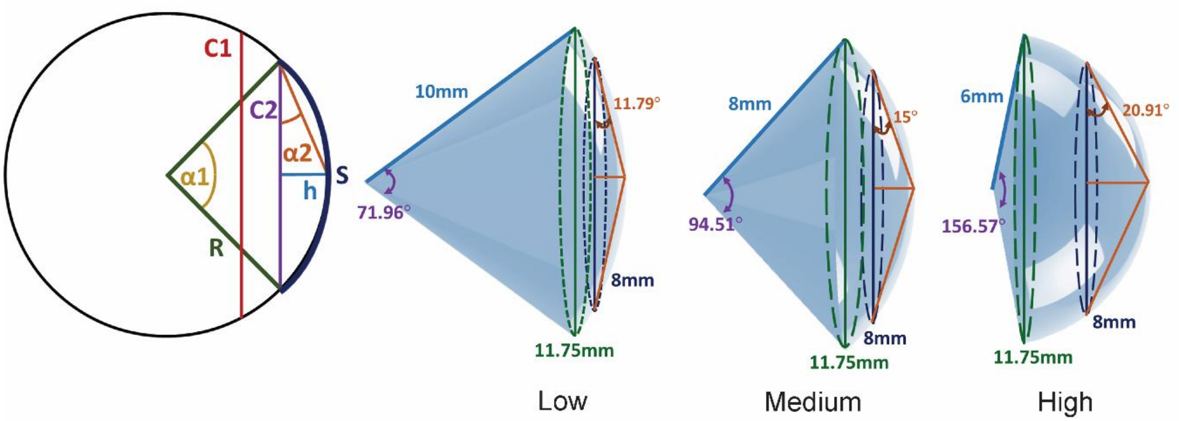

Supplementary Figure 19. The information of three different curvatures; low, medium, and high. Three different curvatures were determined using the circular segment formulas based on the characteristics of the anterior surface of the cornea. To calculate the curvatures, a fixed value of C2 (corneal curvature) was set to 8mm, and C1 (horizontal diameter) was set to 11.75mm. The curvatures were determined based on angles using unit K (diopters = 337.5/r). Three different curvatures were classified; 1) Plana typically has corneal curvature <36D, 2) Normal corneal curvature is 43D, 3) and Keratoconus >45D.

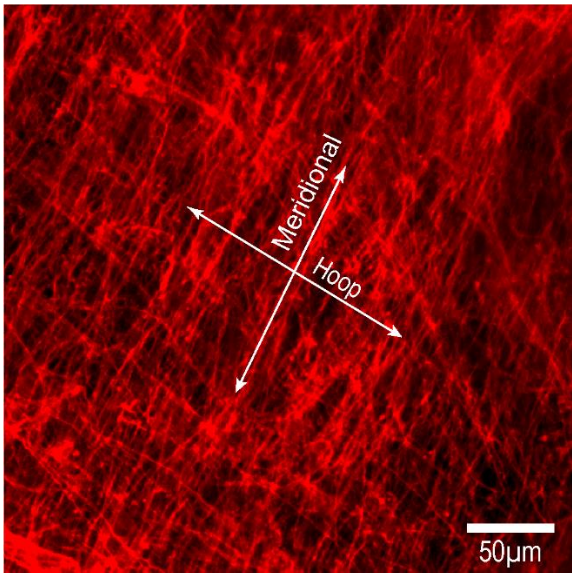

Supplementary Figure 20. Corneal fibroblast cells cultured on curvature for 4 weeks. Representative F-actin image showing stable orthogonal alignment, arising from the interplay of meridional and hoop stresses, and demonstrating long-term maintenance of curvature-induced organization.

Supplementary Table 1. The detailed parameters for three different curvatures with diopters, radius (curve), angle, height, and horizontal diameter.

	Diopters (D)	R (mm)	α1 (°)	α2 (°)	Height (h)	C1 (mm)	C2 (mm)	S (mm)	Area (mm^2)
**Low**	33.75	10	71.96	11.79	0.83	11.75	8	12.56	52.12
**Medium**	42.18	8	94.51	15	1.07	11.75	8	13.20	53.76
**High**	56.25	6	156.57	20.91	1.53	11.75	8	16.40	57.65

Supplementary Table 2. Specific antibodies used in immunostaining to evaluate protein expression.

**1st Antibody**			
**Antigen**	**Host**	**Catalog**	**Manufacturer**
ALDH3A1	rabbit	ab76976	Abcam
a-SMA	mouse	ab7817	Abcam
COL1A1	mouse	MAB3391	Sigma-Aldrich
Vinculin	rabbit	ab129002	Abcam
Vinculin	mouse	53-9777-82	Thermo
pFAK	rabbit	ab81298	Abcam
Rho A	mouse	66733-1	Proteintech
ARPC2	rabbit	ab133315	Abcam
**2nd Antibody**			
**Antigen**	**Host**	**Catalog**	**Manufacturer**
Alexa 594	goat	A-11012	Invitrogen
Alexa 488	goat	SAB4600388	Sigma-Aldrich

Supplementary Table 3. Specific primers are used in quantitative RT-PCR to evaluate gene expression.

Gene	Acession no.	Sequence (5'-3')	Annealing Tm (°C)	Size (bp)
GAPDH	NM_001256799.3	**F**: gatttggtcgtattgggcgc<br>**R**: ttcccgttctcagccttgac	**F:** 59°C<br>**R:** 59°C,	169
COL1A1	NM_000088.4	**F**: gcttcacctacagcgtcact<br>**R**: aagccgaattcctggtctgg	**F:** 59.1°C<br>**R:** 59.1°C	154
COL3A1	NM_000090.4	**F**: agcctggtaagaatggtgcc<br>**R**: tccttgccatcttcgccttt	**F:** 59.1°C<br>**R:** 59°C	103
COL5A1	NM_000093.5	**F**: tgctctttgtctcggaccac<br>**R**: aggtcttcggggtcttcgta	**F:** 59°C<br>**R**: 59°C	187
Lumican	NM_002345.4	**F:** tcctggcattgattggtggt<br>**R**: ttgggtagctttcagggcag	**F:** 58.6°C<br>**R**: 59°C	120
Keratocan	NM_007035.4	**F:** gcctccaagattaccagcca<br>**R**: tgagcactgattcggggaac	**F:** 59.7°C<br>**R**: 60°C	228
ALDH3A1	NM_001330150.2	**F:** atcgtccacatcaccttgca<br>**R**: tcagtgctgggtcatcttgg	**F:** 58.7°C<br>**R**: 58.7°C	189
α-SMA	NM_001406462.1	**F:** aaatgaacgtttccgctgcc<br>**R**: tacatagtggtgccccctga	**F:** 59.1°C<br>**R**: 59°C	165

Supplementary Software 1. A MATLAB code for quantifying fluorescent intensity

```
% Multiple files browse
clear, clc, close all
cd('F:\ Regulation of Corneal Stromal Cell Behavior by Modulating Curvature Using a
Hydraulically Controlled Organ Chip Array\Minju Kim');
list=dir('F:\ Regulation of Corneal Stromal Cell Behavior by Modulating Curvature Using a
Hydraulically Controlled Organ Chip Array\Minju Kim et al\*.tif');
N=size(list,1);
for image_number = 1:N
image_name = list(image_number).name;
f=imread(image_name);
%% Red intensity
r=f(:,:,1);
R_img_gray = im2gray(r);
% Preprocess the image
R_img_smooth = imgaussfilt(R_img_gray, 3);
R_img_thresh = graythresh(R_img_smooth);
R_img_binary = imbinarize(R_img_smooth, R_img_thresh);
% Select the background
R_bg_binary = ~R_img_binary;
% Identify the cells
R_cell_labels = bwlabel(R_img_binary);
% Measure the fluorescent intensity and area of each cell
R_intensities = [];
% R_bg_intensities = [];
R_areas = [];
for i = 1:max(R_cell_labels(:))
R_cell_mask = R_cell_labels == i;
R_cell_intensity = sum(double(R_img_gray(R_cell_mask)));
R_cell_area = sum(R_cell_mask(:));
R_intensities = [R_intensities, R_cell_intensity];
R_areas = [R_areas, R_cell_area];
end
m1= regionprops(R_bg_binary,R_img_gray,'meanintensity');
Mean_bg_R= [m1.MeanIntensity];
CTCF_R = sum(R_intensities)-(mean(Mean_bg_R)*sum(R_areas));
STD_R = std(R_intensities)-(std(Mean_bg_R)*std(R_areas));
%% Green intensity
g=f(:,:,2);
G_img_gray = im2gray(g);
% Preprocess the image
G_img_smooth = imgaussfilt(G_img_gray, 3);
G_img_thresh = graythresh(G_img_smooth);
G_img_binary = imbinarize(G_img_smooth, G_img_thresh);
% Select the background
G_bg_binary = ~G_img_binary;
% Identify the cellsG_cell_labels = bwlabel(G_img_binary);
% Measure the fluorescent intensity and area of each cell
G_intensities = [];
% G_bg_intensities = [];
G_areas = [];
for i = 1:max(G_cell_labels(:))
G_cell_mask = G_cell_labels == i;
G_cell_intensity = sum(double(G_img_gray(G_cell_mask)));
G_cell_area = sum(G_cell_mask(:));
G_intensities = [G_intensities, G_cell_intensity];
G_areas = [G_areas, G_cell_area];
```

```
end
m2= regionprops(G_bg_binary,G_img_gray,'meanintensity');
Mean_bg_G= [m2.MeanIntensity];
CTCF_G = sum(G_intensities)-(mean(Mean_bg_G)*sum(G_areas));
STD_G = std(G_intensities)-(std(Mean_bg_G)*std(G_areas));
%% Dapi cell count
b = f(:,:,3);
%A2 = imadjust(b);
%A3 = imclearborder(A2);
A4 = medfilt2(b);
A4 = imgaussfilt(A4,3);
A4 = im2bw(A4);%,graythresh(A4));
A5 = imregionalmax(A4);%,6);
% Label and count connected components
[L, Ne]=bwlabel(double(A5));
prop=regionprops(L,'Area','Centroid');
total=0;
for n=1:size(prop,1) %For 1 to Total number of dapi
cell=prop(n).Centroid;
if prop(n).Area>1
total=total+1;
else
end
end
num_cell = total-1;
%% calculate
redmean = CTCF_R;
greenmean = CTCF_G;
Std_R=STD_R;
Std_G=STD_G;
%disp(['Red Mean intensity= ' num2str(Mean_inten_R/no)])
%disp(['Green Mean intensity= ' num2str(Mean_inten_G/no)])
image_name_list(image_number,:) = convertCharsToStrings(image_name);
red_fluorescence_list(image_number,:) = convertCharsToStrings(redmean);
green_fluorescence_list(image_number,:) = convertCharsToStrings(greenmean);num_cell_list(image_number,:) =
convertCharsToStrings(num_cell);
red_mean_intensity_list(image_number,:) =
convertCharsToStrings(redmean/num_cell);
green_mean_intensity_list(image_number,:) =
convertCharsToStrings(greenmean/num_cell);
red_stdev_list(image_number,:) = convertCharsToStrings(Std_R);
green_stdev_list(image_number,:) = convertCharsToStrings(Std_G);
red_meanstdev_list(image_number,:) = convertCharsToStrings(Std_R/num_cell);
green_meanstdev_list(image_number,:) =
convertCharsToStrings(Std_G/num_cell);
end
C =
table(image_name_list,red_fluorescence_list,green_fluorescence_list,num_cell_list,red_mean_in
tensity_list,green_mean_intensity_list,red_stdev_list,green_stdev_list,red_meanstdev_list,green_
meanstdev_list)
C.Properties.VariableNames = ["File Name", "Red fluorescence", "Green fluorescence", "Cell
Count Value", "Red Mean Intensity", "Green Mean Intensity", "Red STDEV", "Green STDEV",
"Red mean STDEV", "Green mean STDEV"]
filename = 'quantitative fluorescent analysis.xlsx';
writetable(C,filename)
```

References

1. Kim TK, Kim JK, Jeong OC. Measurement of nonlinear mechanical properties of PDMS elastomer. *Microelectronic Engineering* **88**, 1982-1985 (2011).
2. Ford AJ, Rajagopalan P. Extracellular matrix remodeling in 3D: implications in tissue homeostasis and disease progression. *Wiley Interdisciplinary Reviews: Nanomedicine and Nanobiotechnology* **10**, e1503 (2018).
3. Sip CG, Folch A. Stable chemical bonding of porous membranes and poly (dimethylsiloxane) devices for long-term cell culture. *Biomicrofluidics* **8**, (2014).
4. Muthusubramaniam L, Peng L, Zaitseva T, Paukshto M, Martin GR, Desai TA. Collagen fibril diameter and alignment promote the quiescent keratocyte phenotype. *J Biomed Mater Res A* **100**, 613-621 (2012).
5. Karamichos D, Lakshman N, Petroll WM. Regulation of corneal fibroblast morphology and collagen reorganization by extracellular matrix mechanical properties. *Invest Ophthalmol Vis Sci* **48**, 5030-5037 (2007).
6. Mawaki A, Nakatani T, Sugama J, Konya C. Relationship between the distribution of myofibroblasts, and stellar and circular scar formation due to the contraction of square and circular wound healing. *Anatomical Science International* **82**, 147-155 (2007).
7. Shinde AV, Humeres C, Frangogiannis NG. The role of alpha-smooth muscle actin in fibroblast-mediated matrix contraction and remodeling. *Biochim Biophys Acta Mol Basis Dis* **1863**, 298-309 (2017).
8. Hinz B, Dugina V, Ballestrem C, Wehrle-Haller B, Chaponnier C. Alpha-smooth muscle actin is crucial for focal adhesion maturation in myofibroblasts. *Mol Biol Cell* **14**, 2508-2519 (2003).
9. Chen J*, et al.* Stiffness-dependent dynamic effect of inflammation on keratocyte phenotype and differentiation. *Biomed Mater* **18**, (2023).
