# Supplementary material for: Regulation of corneal stromal cell behavior by modulating curvature using a hydraulically-controlled organ chip array
Source: Nat Commun. 2025 Nov 12;16:9944. doi: 10.1038/s41467-025-64889-8 (PMC12612184; doi:10.1038/s41467-025-64889-8)
Supplement: Supplementary file 2 — Reporting Summary [file 41467_2025_64889_MOESM2_ESM.pdf]

nature portfolio

Corresponding author(s): NCOMMS-24-10590B

Last updated by author(s): 09142025

Reporting Summary

Nature Portfolio wishes to improve the reproducibility of the work that we publish. This form provides structure for consistency and transparency in reporting. For further information on Nature Portfolio policies, see our Editorial Policies and the Editorial Policy Checklist.

Statistics

For all statistical analyses, confirm that the following items are present in the figure legend, table legend, main text, or Methods section.

n/a	Confirmed	
☐	☒	The exact sample size (*n*) for each experimental group/condition, given as a discrete number and unit of measurement
☐	☒	A statement on whether measurements were taken from distinct samples or whether the same sample was measured repeatedly
☐	☒	The statistical test(s) used AND whether they are one- or two-sided<br>*Only common tests should be described solely by name; describe more complex techniques in the Methods section.*
☒	☐	A description of all covariates tested
☐	☒	A description of any assumptions or corrections, such as tests of normality and adjustment for multiple comparisons
☐	☒	A full description of the statistical parameters including central tendency (e.g. means) or other basic estimates (e.g. regression coefficient) AND variation (e.g. standard deviation) or associated estimates of uncertainty (e.g. confidence intervals)
☐	☒	For null hypothesis testing, the test statistic (e.g. *F*, *t*, *r*) with confidence intervals, effect sizes, degrees of freedom and *P* value noted<br>*Give P values as exact values whenever suitable.*
☒	☐	For Bayesian analysis, information on the choice of priors and Markov chain Monte Carlo settings
☒	☐	For hierarchical and complex designs, identification of the appropriate level for tests and full reporting of outcomes
☒	☐	Estimates of effect sizes (e.g. Cohen's *d*, Pearson's *r*), indicating how they were calculated

Our web collection on statistics for biologists contains articles on many of the points above.

Software and code

Policy information about availability of computer code

Data collection	Fluorescent images were acquired using a Nikon Eclipse Ti microscope (Nikon Instruments, Tokyo, Japan) with NIS Elements Advanced Research software. Image analysis was performed with the OrientationJ plug-in (Biomedical Imaging Group, EPFL, Switzerland) in ImageJ (NIH, Bethesda, MD, USA), and custom MATLAB scripts (R2022b, MathWorks, Natick, MA, USA) were used to quantify mean fluorescence intensity. Absorbance for the cell viability assay was measured using a microplate reader (Epoch 2, BioTek, Winooski, VT, USA). Total RNA purity for qRT-PCR was assessed by a Nanodrop spectrophotometer (ND-1000, Thermo Fisher Scientific, Waltham, MA, USA). cDNA synthesis was performed using a Veriti 96-well thermal cycler (Applied Biosystems, Foster City, CA, USA), and quantitative PCR was conducted on a QuantStudio 12K Flex Real-Time PCR system (Applied Biosystems) with SYBR Green detection. For RNA sequencing, total RNA quality was assessed by NanoDrop and Bioanalyzer. Libraries were prepared with the NEBNext Ultra II RNA Library Prep Kit (New England Biolabs, Ipswich, MA, USA) using poly(A) selection and sequenced on an Illumina NovaSeq X Plus platform (Illumina, San Diego, CA, USA), generating ~40–60 million 150 bp paired-end reads per sample.
Data analysis	Imaging data were analyzed using custom MATLAB scripts (R2022b, MathWorks) to quantify nuclear counts and mean RGB fluorescence intensity, with corrected total cell fluorescence (CTCF) used for intensity calculation. qRT-PCR data were analyzed by relative quantification using the 2^(-ΔΔCt) method. RNA-seq reads were quality-checked with FastQC, trimmed with Trimmomatic, aligned to the human reference genome (GRCh38) using STAR, and quantified with featureCounts. Differential expression analysis was performed with DESeq2, applying Benjamini–Hochberg correction, and enrichment analyses were conducted with clusterProfiler in R. Graphical representation and statistical analyses were performed using Excel (Microsoft), OriginPro 2023b (OriginLab), and SPSS 29.0 (IBM).

For manuscripts utilizing custom algorithms or software that are central to the research but not yet described in published literature, software must be made available to editors and reviewers. We strongly encourage code deposition in a community repository (e.g. GitHub). See the Nature Portfolio guidelines for submitting code & software for further information.

Data

Policy information about availability of data

All manuscripts must include a data availability statement. This statement should provide the following information, where applicable:

- Accession codes, unique identifiers, or web links for publicly available datasets
- A description of any restrictions on data availability
- For clinical datasets or third party data, please ensure that the statement adheres to our policy

All specific information supporting the main findings of this study is provided in the Supplementary Information. The custom MATLAB code used for quantitative fluorescence analysis is publicly available at Code Ocean (https://doi.org/10.24433/CO.7180087.v1). The raw RNA sequencing data generated in this study have been deposited in the NCBI Gene Expression Omnibus (GEO) under accession code GSEXXXXXX. All other relevant data are included in the main text and Supplementary Information and are provided as Source Data.

Research involving human participants, their data, or biological material

Policy information about studies with human participants or human data. See also policy information about sex, gender (identity/presentation), and sexual orientation and race, ethnicity and racism.

Reporting on sex and gender	N/A
Reporting on race, ethnicity, or other socially relevant groupings	N/A
Population characteristics	N/A
Recruitment	N/A
Ethics oversight	N/A

Note that full information on the approval of the study protocol must also be provided in the manuscript.

Field-specific reporting

Please select the one below that is the best fit for your research. If you are not sure, read the appropriate sections before making your selection.

[x] Life sciences [ ] Behavioural & social sciences [ ] Ecological, evolutionary & environmental sciences

For a reference copy of the document with all sections, see nature.com/documents/nr-reporting-summary-flat.pdf

Life sciences study design

All studies must disclose on these points even when the disclosure is negative.

Sample size	Sample size was defined based on the average radius of human corneal curvature. All curvature values in this study were calculated using circular segment formulas. Curvature angle and height were measured at least five times. For cell behavior experiments on the curvature chip, a minimum of three independent biological replicates were performed to obtain mean values and standard deviations. The number of analyzed samples (n) is indicated in the figure legends of the manuscript.
Data exclusions	No data were excluded from the analyses. All samples and measurements that were collected were included in the reported results.
Replication	All experiments were performed at least three independent times to verify reproducibility. For imaging analysis, total expression levels were quantified from whole samples using custom MATLAB code, rather than partial image selection.
Randomization	Corneal curvature chips were randomly assigned to experimental groups. Samples designated for each biological analysis were also randomly distributed among groups.
Blinding	Blinding was not required, as samples were randomly assigned to groups and analyses were performed without bias.

Reporting for specific materials, systems and methods

We require information from authors about some types of materials, experimental systems and methods used in many studies. Here, indicate whether each material, system or method listed is relevant to your study. If you are not sure if a list item applies to your research, read the appropriate section before selecting a response.

Materials & experimental systems

n/a	Involved in the study	
☐	☒	Antibodies
☐	☒	Eukaryotic cell lines
☒	☐	Palaeontology and archaeology
☒	☐	Animals and other organisms
☒	☐	Clinical data
☒	☐	Dual use research of concern
☒	☐	Plants

Methods

n/a	Involved in the study	
☒	☐	ChIP-seq
☒	☐	Flow cytometry
☒	☐	MRI-based neuroimaging

Antibodies

Antibodies used	Primary-TRITC Rhodamine phalloidin (P1951, Sigma-Aldrich, diluted 1:100), mouse anti-collagen type 1 (MAB3391, diluted 1:100), rabbit anti-vinculin (ab129002, Abcam, diluted 1:100), mouse anti-vinculin (53-9777-82, Thermo, diluted 1:100), rabbit anti-pFAK (ab81298, Abcam, diluted 1:400), rabbit anti- ALDH3A1 (ab76976, Abcam, diluted 1:100), mouse anti-α-SMA (ab7817, Abcam, diluted 1:100), and secondary-goat anti-mouse Alexa Fluor 488 (SAB4600388, Sigma-Aldrich, diluted 1:200) and goat anti-rabbit Alexa Fluor 594 (A-11012, Invitrogen, diluted 1:1000). All detailed information was specified on Supplementary Table 2.
Validation	All antibodies used in this study were sourced from commercial suppliers and have been previously reported in other research papers. We based our usage on the information detailed in the datasheets provided by the manufacturers. Additionally, we verified the effectiveness of each antibody on cells cultured in commercial wells independently of the curvature chip. We confirmed the results obtained from the flat group on the chip were consistent with those from experiments conducted using standard culture plates.

Eukaryotic cell lines

Policy information about cell lines and Sex and Gender in Research

Cell line source(s)	Primary human corneal keratocytes (HCK, 6520, ScienCell, USA) were purchased from a commercial supplier (ScienCell, USA). All culture protocols were conducted in accordance with the instructions provided by the manufacturer.
Authentication	N/A
Mycoplasma contamination	All cells were tested, and no mycoplasma contamination was detected.
Commonly misidentified lines (See ICLAC register)	No commonly misidentified cell lines were used in this study.

Plants

Seed stocks	N/A
Novel plant genotypes	N/A
Authentication	N/A
